# Supplementary material for: Dual protein kinase and nucleoside kinase modulators for rationally designed polypharmacology
Source: Nat Commun. 2017 Nov 10;8:1420. doi: 10.1038/s41467-017-01582-5 (PMC5681654; doi:10.1038/s41467-017-01582-5)
Supplement: Supplementary file 1 — Supplementary Information [file 41467_2017_1582_MOESM1_ESM.pdf]

SUPPLEMENTARY NOTES

Supplementary Note 1. Synthesis and characterisation of masitinib-NH_2-LC-biotin and DI-39.

General Methods. All solvents were purified according to standard procedures, and the reagents commercially available were used as received. Separation by column chromatography was performed using SDS Kieselgel (70-230 mesh). ^1H and ^{13}C NMR spectra were recorded by using a Brucker Avance III - 600MHz or AC250 spectrometer. Chemical shifts, δ, are reported in ppm and coupling values, *J*, in hertz. Abbreviations for peaks are, s: singlet, d: doublet, t: triplet, and m: multiplet. Reaction monitoring and purity of compounds were recorded by using analytical Agilent Infinity high performance liquid chromatography (Column Zorbax SB-C18 1.8 μM (2.1x50 mm); Mobile phase (A: 0.1% FA H_2O, B: 0.1% FA MeCN, Time/%B 0/10, 4/90, 7/90, 9/10, 10/10); Flow rate 0.3 ml min^{-1}; Diluent MeOH) with DAD at 230 nM. All tested compounds yielded data consistent with a purity of ≥ 95%. Low-resolution mass spectra were obtained with Agilent SQ G6120B mass spectrometer in positive electrospray mode. High-resolution mass spectra were performed at the Spectropole Analytical Laboratory of the Aix-Marseille University, Marseille, using a Waters SYNAPT G2 HDMS mass spectrometer. Compound DI-39 was prepared as previously reported[1].

Synthesis of *N*-[6-oxo-6-[[2-[4-[[4-[[[4-methyl-3-[[4-(3-pyridinyl)-2-thiazolyl]amino]phenyl]amino]carbonyl]phenyl]methyl]-1-piperazinyl]ethyl]amino]hexyl]-hexahydro-2-oxo-[3aS,4S,6aR]-1*H*-thieno[3,4-d]imidazole-4-pentanamide (masitinib-NH_2-LC-biotin). Under argon, at room temperature, to a solution of 4-[[4-(2aminoethyl)-1-piperazinyl]methyl]-*N*-[4-methyl-3-[[4-(3-pyridinyl)-2-thiazolyl]amino]phenyl]benzamide (13 mg, 0.0246 mmol) in dimethylformamide (2 ml) was added in solid fraction *N*-[6-[(2,5-dioxo-1-pyrrolidinyl)oxy]-6-oxohexyl]hexahydro-2-oxo-(3aS,4S,6aR)-1*H*-thieno[3,4-d]imidazole-4-pentanamide (NHS-LC-Biotin) (12.3 mg, 0.0271 mmol). The resulting mixture was stirred for 2 days and the solvent was distilled off under reduced pressure. The residue was purified by column chromatography, gradient of eluent CH_2Cl_2:MeOH:NH_3 (from 10:2:0 to 10:2:0.5 v/v) to afford the corresponding biotinylated masitinib derivative (17 mg, 81%) as a yellow powder. TLC (MeOH): R_f=0.51; ^1H NMR (600 MHz, CD_3OD): δ 9.11 (s, 1H), 8.53 (s, 1H), 8.42 (d, *J* = 4.8 Hz, 1H), 8.36 (d, *J* = 8.1 Hz, 1H),

7.97 (d, J = 8.1 Hz, 2H), 7.55 (d, J = 8.1 Hz, 2H), 7.45 (dd, J = 4.8 and 8.1 Hz, 1H), 7.31 (dd, J = 2.0 and 8.1 Hz, 1H), 7.24-7.23(m, 2H), 4.47 (dd, J = 4.9 and 7.8 Hz, 1H), 4.28 (dd, J = 4.6 and 7.8 Hz, 1H), 3.88 (s, 2H), 3.46 (t, J = 5.7 Hz, 2H), 3.20-2.68 (m, 16H), 2.34 (s, 3H), 2.23 (t, J = 7.5 Hz, 2H), 2.18 (t, J = 7.3 Hz, 2H) and 1.75-1.29 (m, 12H); ^{13}C NMR (150 MHz, MeOD): δ 177.94, 176.84, 175.77, 169.23, 169.09, 166.93, 149.59, 149.38, 148.70, 141.61, 139.40, 136.29, 133.65, 132.79, 131.85, 129.92, 127.71, 126.15, 118.73, 116.37, 106.50, 64.23, 62.88, 62.49, 58.65, 57.86, 53.83, 52.78, 41.92, 40.99, 37.68, 37.65, 31.00, 30.59, 30.36, 28.38, 27.79, 27.23, 27.15, 18.58; LCMS $C_{45}H_{58}N_{10}O_4S_2$ R_t=6.166 min, ESI-MS (m/z): 866.5 $[M+H]^+$, 433.8 $[M+2H]^{2+}$, 289.7 $[M+3H]^{3+}$, purity >98%; HRMS (m/z): $[M+H]^+$ calculated for $C_{45}H_{59}N_{10}O_4S_2$, 867.4157; found 867.4162.

***N*-(2-(5-(4-(((4,6-Diaminopyrimidin-2-yl)thio)methyl)-5-propylthiazol-2-yl)-2-methoxyphenoxy)ethyl)methanesulfonamide (DI-39).** Purified by column chromatography, eluent CH_2Cl_2:MeOH (10:1 v/v) as a light yellow solid. TLC (CH_2Cl_2:MeOH, 10:1 v/v): R_f=0.29; ^{1}H NMR (250 MHz, CD_3OD) δ 7.51 (d, J = 2.1 Hz, 1H), 7.41 (dd, J = 8.5 and 2.1 Hz, 1H), 7.02 (d, J = 8.5 Hz, 1H), 5.33 (s, 1H), 4.49 (s, 2H), 4.15 (t, J = 5.2 Hz, 2H), 3.88 (s, 3H), 3.53 (t, J = 5.2 Hz, 2H), 3.06 (s, 3H), 2.92 (t, J = 7.4 Hz, 2H), 1.67 (sex, J = 7.4 Hz, 2H) and 0.98 (t, J = 7.4 Hz, 3H). LCMS $C_{21}H_{28}N_6O_4S_3$ R_t=6.370 min, ESI-MS (m/z): 524.5 $[M+H]^+$, 262.9 $[M+2H]^{2+}$, purity >95%.

SUPPLEMENTARY FIGURES

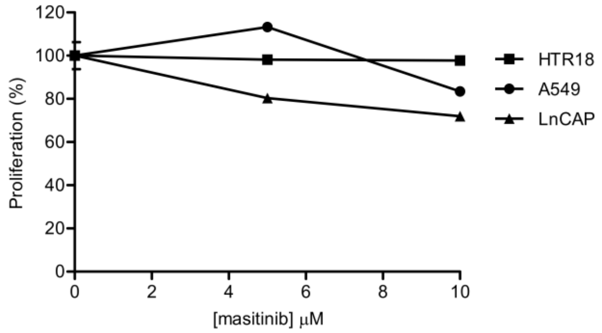

Supplementary Figure 1. Anti-proliferative activity of masitinib monotherapy on HRT18, A549 and LnCAP cell lines. HRT18 (colon), A549 (lung), and LNCaP (prostate) cancer cell lines were tested in proliferation assays for response to masitinib (0 to 10 μM). The experiments were performed in triplicates and data are presented as the mean ± s.d.

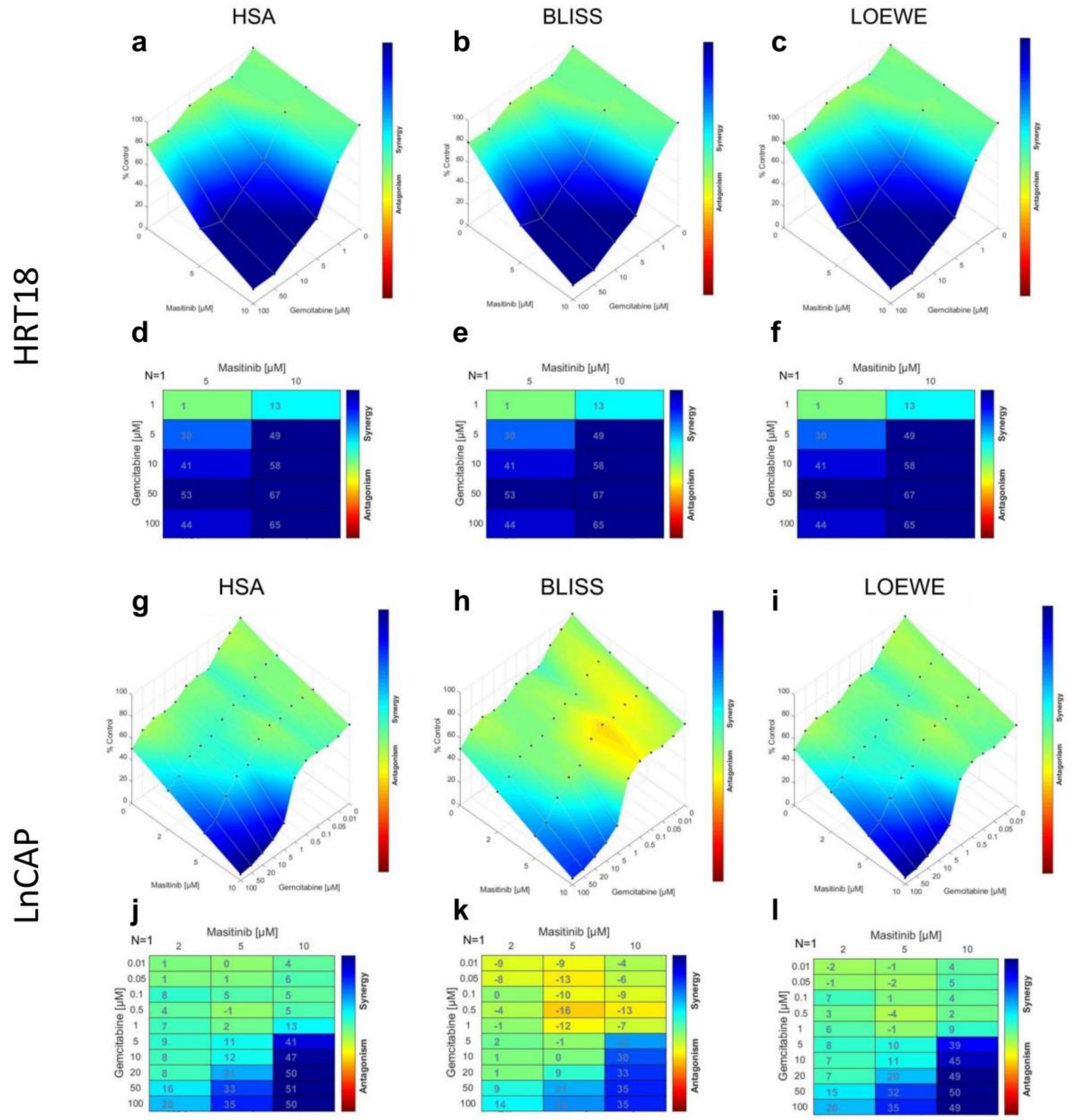

Supplementary Figure 2. Synergy analysis by Combenefit[2]. Data from combination treatments with gemcitabine and masitinib in HRT18 (**a-f**) and LnCAP (**g-l**) cell lines were processed using three synergy reference models: Highest Single Agent (HSA) model (**a**, **d**, **g**, **j**), Bliss independence model (**b**, **e**, **h**, **k**) and Loewe additivity model (**c**, **f**, **i**, **l**). 3D plots and matrix are presented for each analysis. The synergy is expressed as a colour scale ranging from blue (synergy) to red (antagonism).

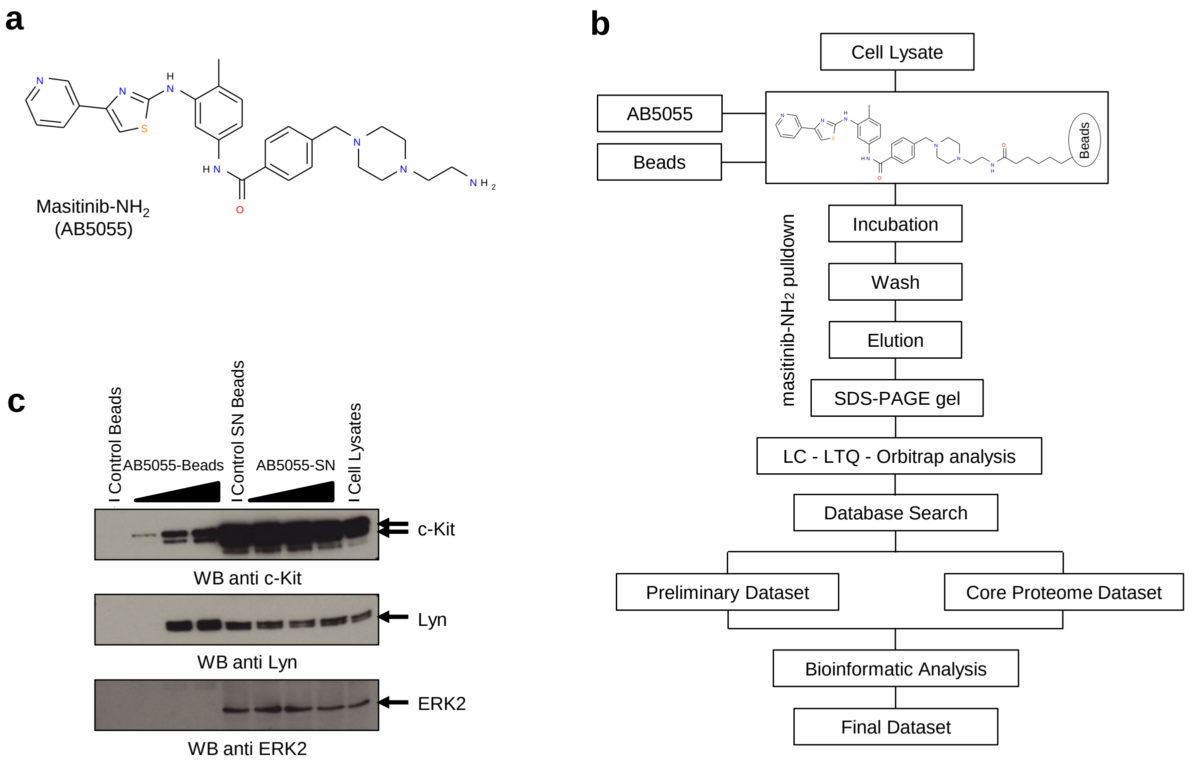

Supplementary Figure 3. Strategy of dCK identification. (**a**) Structure of the NH_2-modified version of masitinib (AB5055). (**b**) Schematic representation of the different steps of chemical proteomics experiments and core proteome analysis (adapted from Rix *et al*, 2007)[3]. (**c**) Confirmation of the activity and specificity of AB5055 interaction on two of its main tyrosine kinase targets (c-Kit and Lyn) using western blot with specific antibodies. ERK2 antibody was used as negative control for pull down experiments.

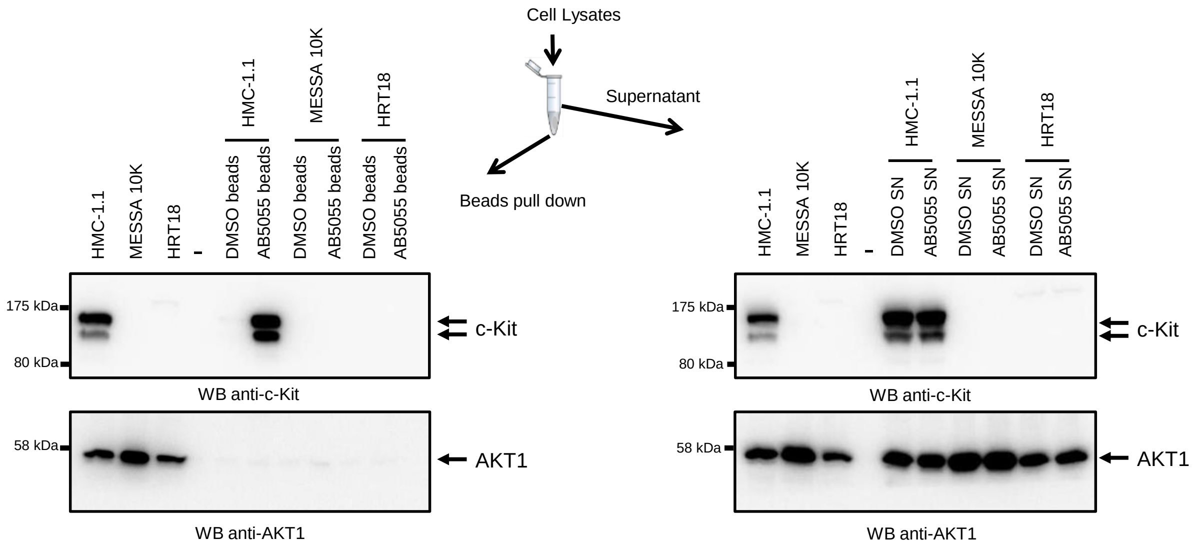

Supplementary Figure 4. Validation of AB5055/c-Kit interaction by pull-down experiments. Confirmation of the activity and specificity of interaction of AB5055 on its main tyrosine kinase target c-Kit on three cell lines lysates (HMC-1.1, MESSA 10K and HRT18) by pull down and western blot with specific antibody. HMC-1.1 and Messa 10K cell lysates were taken respectively as positive and negative control for dCK expression. AKT1 antibody was used as negative control for pull down experiments.

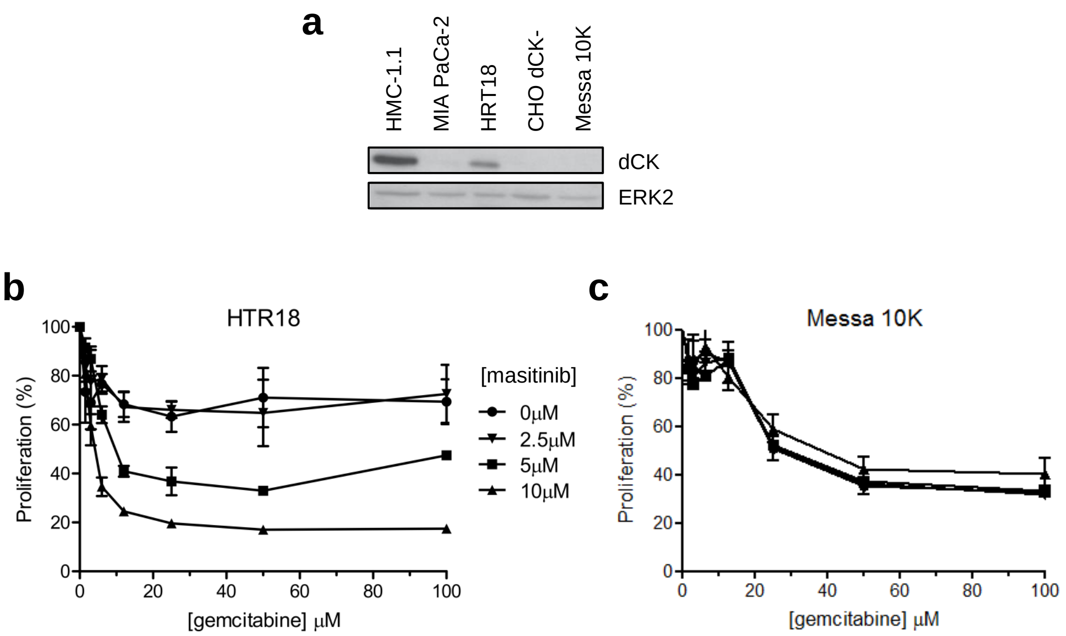

Supplementary Figure 5. Effect of a masitinib/gemcitabine combination treatment on cell lines with different dCK expression profiles. (**a**) Level of expression of dCK in different cell lines determined by western blot analysis. dCK was expressed in HMC-1.1, and partially expressed in HRT18. The protein was not expressed in Messa 10K and CHO dCK-. ERK2 was used as the loading control. Proliferation assays of HRT18 (**b**), and Messa 10K (**c**) cell lines in the presence and absence of masitinib at different concentrations (0, 2.5 µM, 5 µM, 10 µM) and gemcitabine at different concentrations (0 to 100 µM). The combination of gemcitabine and masitinib reduced the viable HRT18 cells whereas the same combination has no effect on the proliferation of Messa 10K gemcitabine-resistant cell line. The experiments were performed in triplicates and the data are presented as the mean ± s.d.

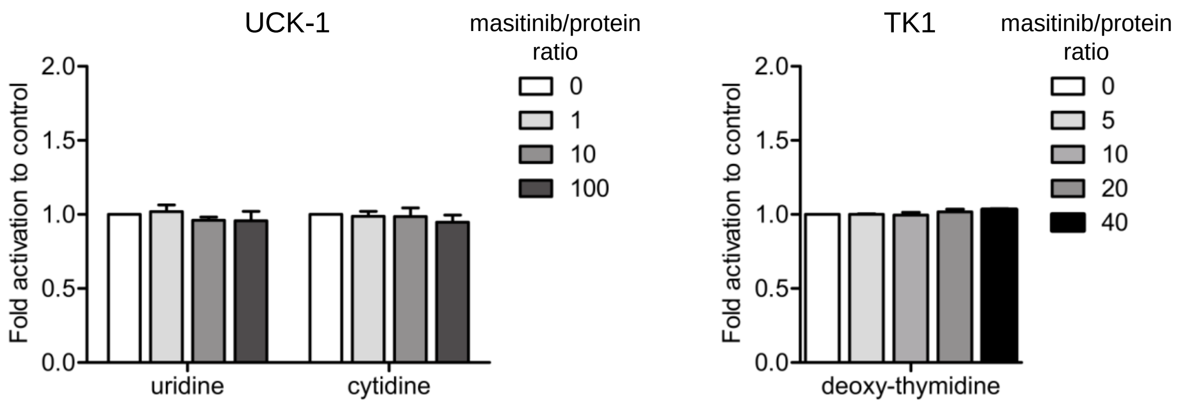

Supplementary Figure 6. Masitinib effect on other nucleoside kinases. Enzymatic reaction of UCK-1 and TK1 in the presence of various concentrations of masitinib (masitinib/protein ratios: 0, 1, 10, 100, and 1, 5, 10, 20, 40, respectively). Unlike dCK, masitinib didn't produce an enzymatic modulation on these nucleoside kinases. The experiments were performed in triplicates and data are presented as the mean ± s.d.

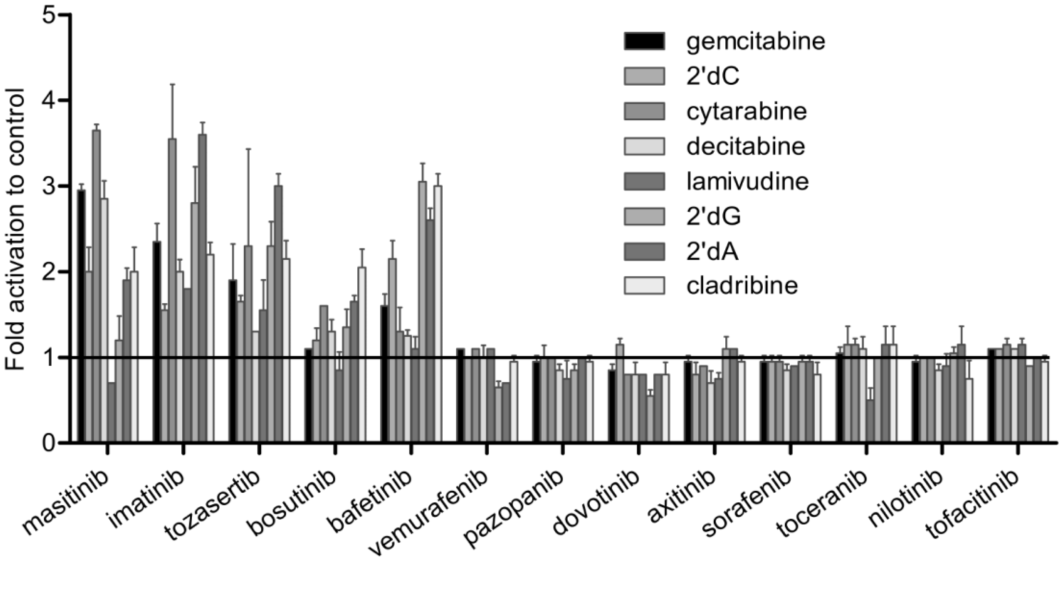

Supplementary Figure 7. Analysis of the effect of masitinib and other protein kinase inhibitors on dCK enzymatic phosphorylation of substrates in the presence of UTP. Protein kinase inhibitors were investigated to evaluate their effect on substrate phosphorylation by dCK in the presence of UTP (inhibitor/protein ratio: 22). The experiments were performed in triplicates and data are presented as the mean ± s.d.

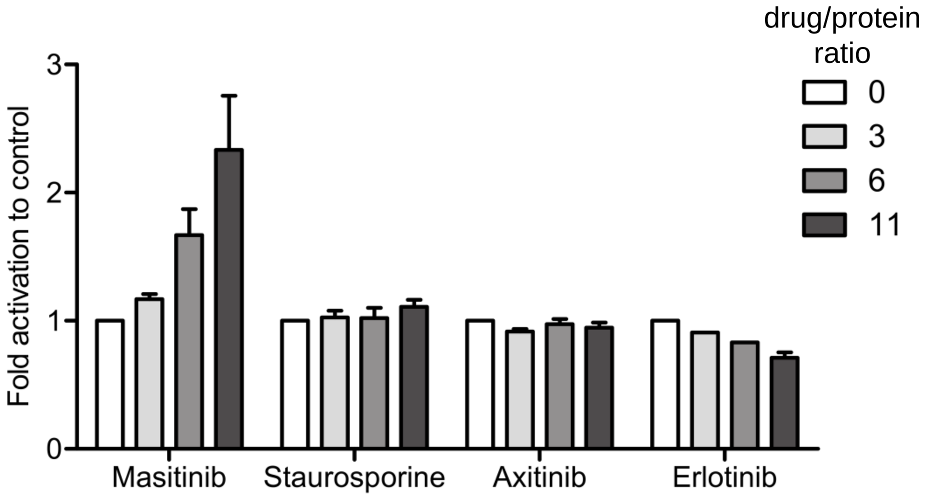

Supplementary Figure 8. Effect of masitinib, saturosporine, axitinib, and erlotinib on gemcitabine enzymatic phosphorylation by dCK. Staurosporine, axitinib and erlotinib have no activation effect on direct dCK enzymatic monophosphorylation of gemcitabine, unlike masitinib. Velocity was standardized with respect to the drug free control and the level of activation was defined as the ratio between the velocity at a given drug concentration and the velocity in the absence of drug (drug/protein ratios: 0, 3, 6, 11). The experiments were performed in triplicates and data are presented as the mean ± s.d.

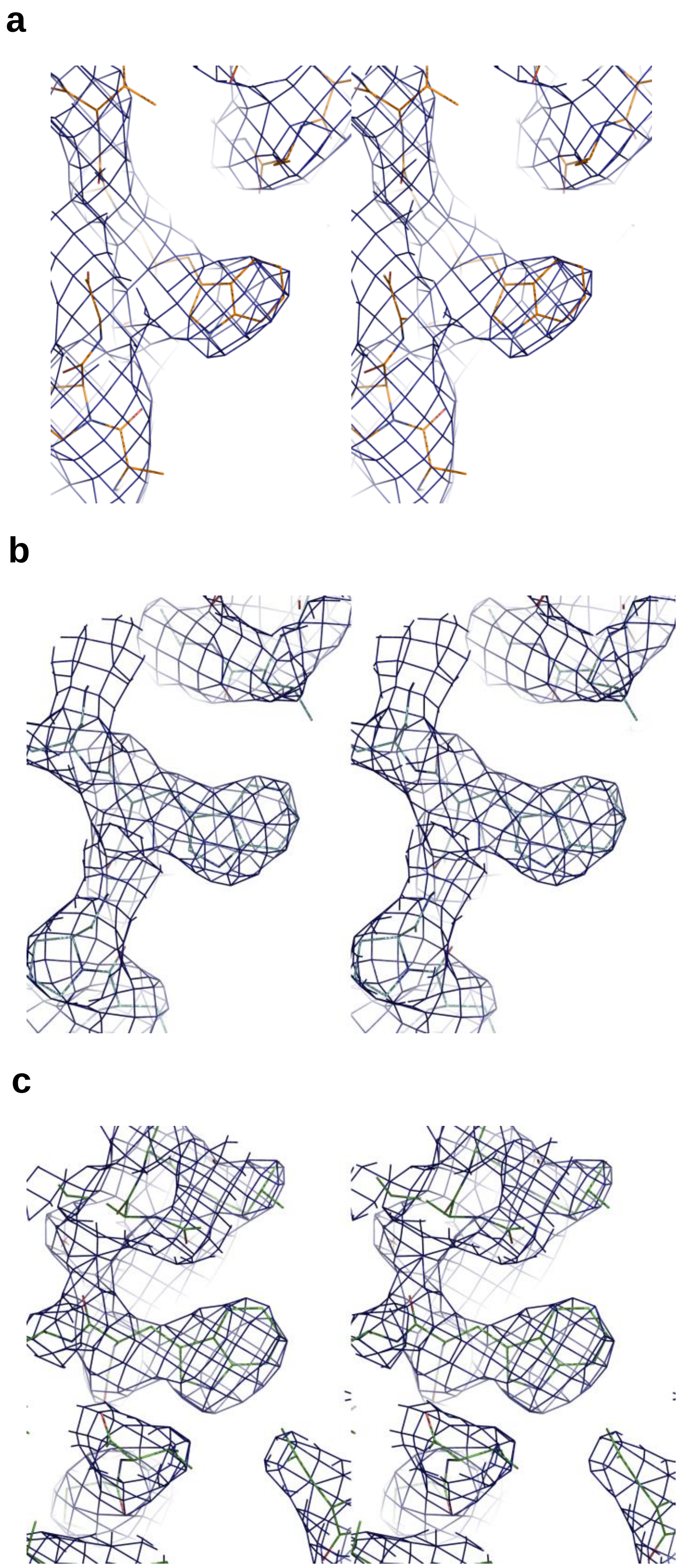

Supplementary Figure 9. Stereo images of the electron density of dCK crystal structures. Stereo image of the 2Fo-Fc electron density map contoured at 1σ around the residue Trp48 for the structure of dCK apo (**a**), dCK in complex with masitinib (**b**) and dCK in complex with imatinib (**c**).

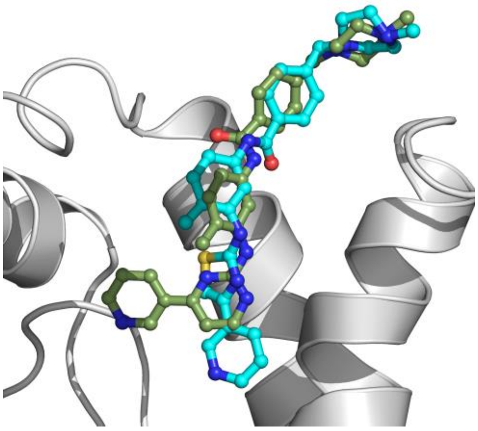

Supplementary Figure 10. Overlay of dCK crystal structures in complex with masitinib or imatinib. Detail of the overlay of masitinib (cyan) and imatinib (green) in the substrate binding site (cartoon representation).

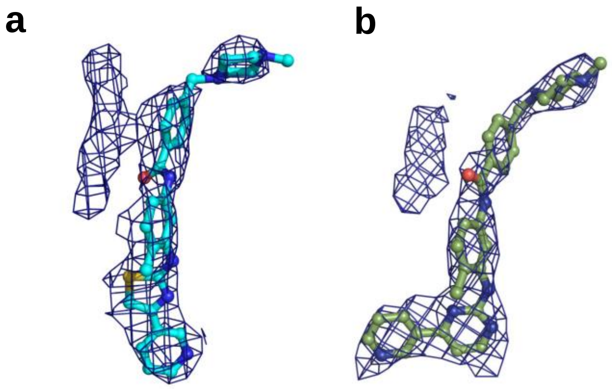

Supplementary Figure 11. 2mFo-DFc omit map around the TKIs in crystal structures of dCK in complex with masitinib or imatinib. 2mFo-DFc omit map contoured at 0.9σ around masitinib (cyan) (**a**) and imatinib (green) (**b**). A supplementary electron density is present at the top of the cavity for both complexes, likely corresponding to a second molecule of compound interacting weakly with the first one.

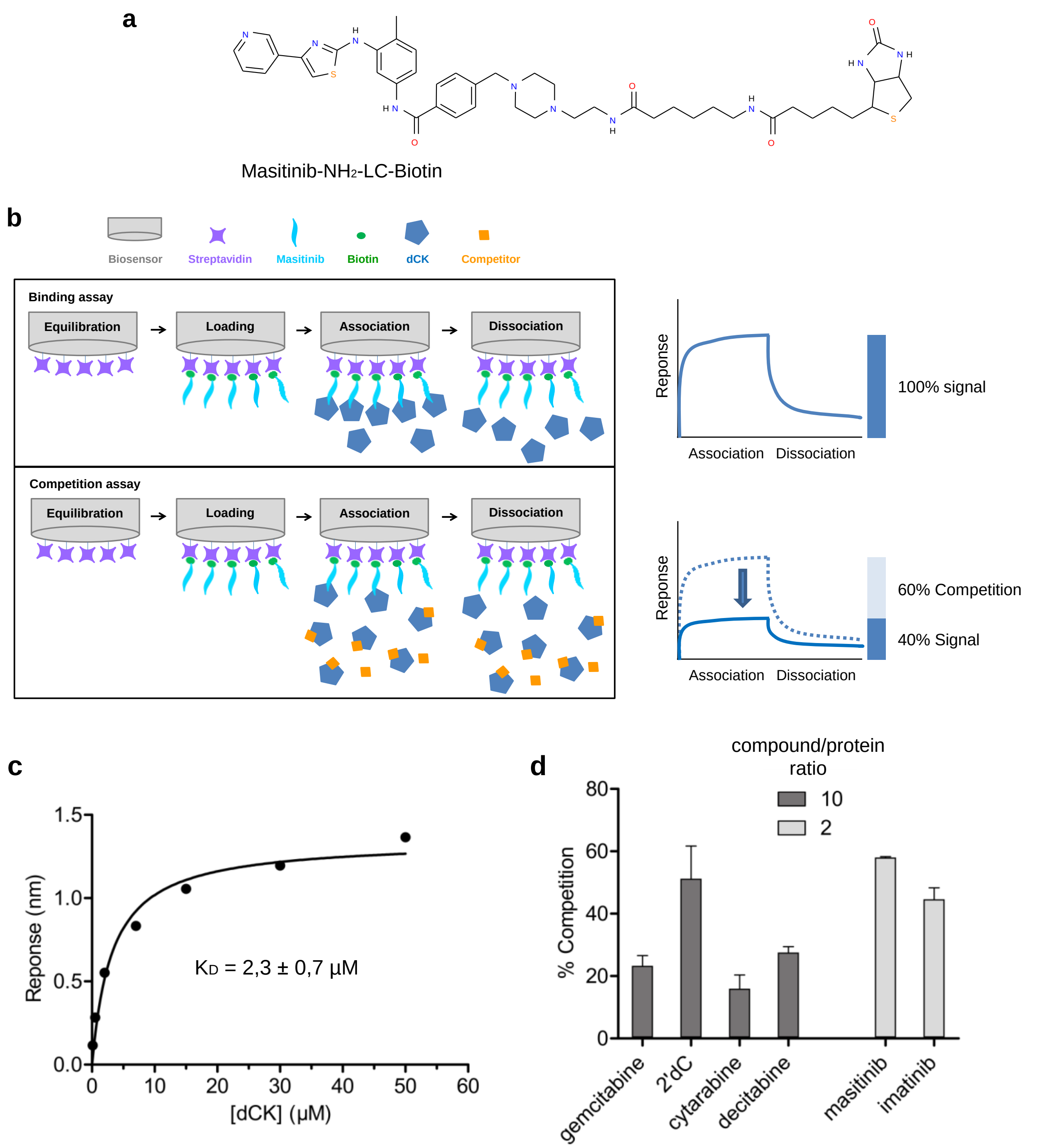

Supplementary Figure 12. dCK binding to substrates and kinase inhibitors determined by Bio-Layer Interferometry (BLI). (**a**) Structure of the biotin-modified version of masitinib. (**b**) Schematic representation of binding experiments using SSA biosensors. (**c**) dCK (0.1–50 µM) binding to streptavidin-coated biosensors loaded with a conjugated-biotin version of masitinib. The dissociation constant (K_D) was determined using data at equilibrium (response at the end of the association step) using steady state analysis. (**d**) Competitions assays: binding of dCK (10 µM) to fixed masitinib was measured in the presence or the absence of competitors (100 µM for substrates (gemcitabine, 2'dC, decitabine, aracytine) and 20 µM for kinase inhibitors (masitinib and imatinib)) and standardized with respect to the competitor free control. Each experiment was performed three times and data are presented as the mean ± s.d.

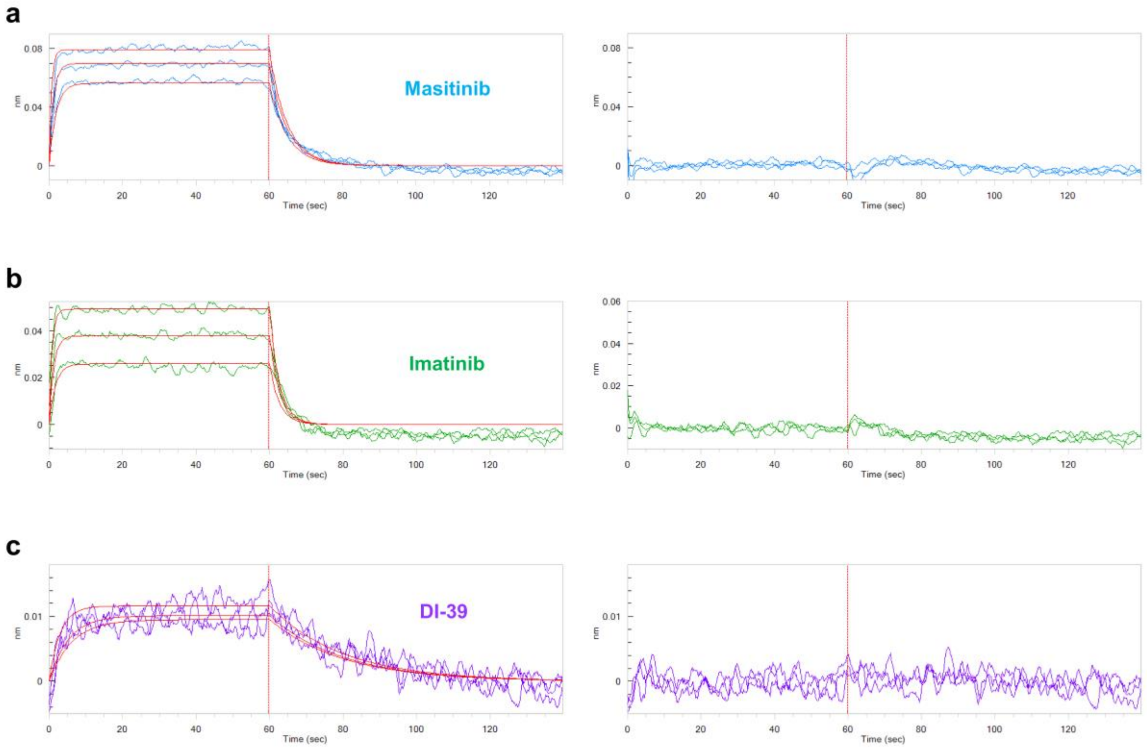

Supplementary Figure 13. Kinetic analysis for dCK binding to masitinib, imatinib and DI-39 by Bio-Layer Interferometry (BLI). Representative BLI sensorgrams display the processed binding data and the fitted binding curve (red) for analysed biosensors (masitinib (**a**), imatinib (**b**), and DI-39 (**c**)) in left panel. For each experiment, the right panel displays the residual view, representing the difference between the raw binding data and the fitted curve.

a

^{1}H NMR (600 MHz, CD_3OD) Masitinib-NH_2-LC-biotin

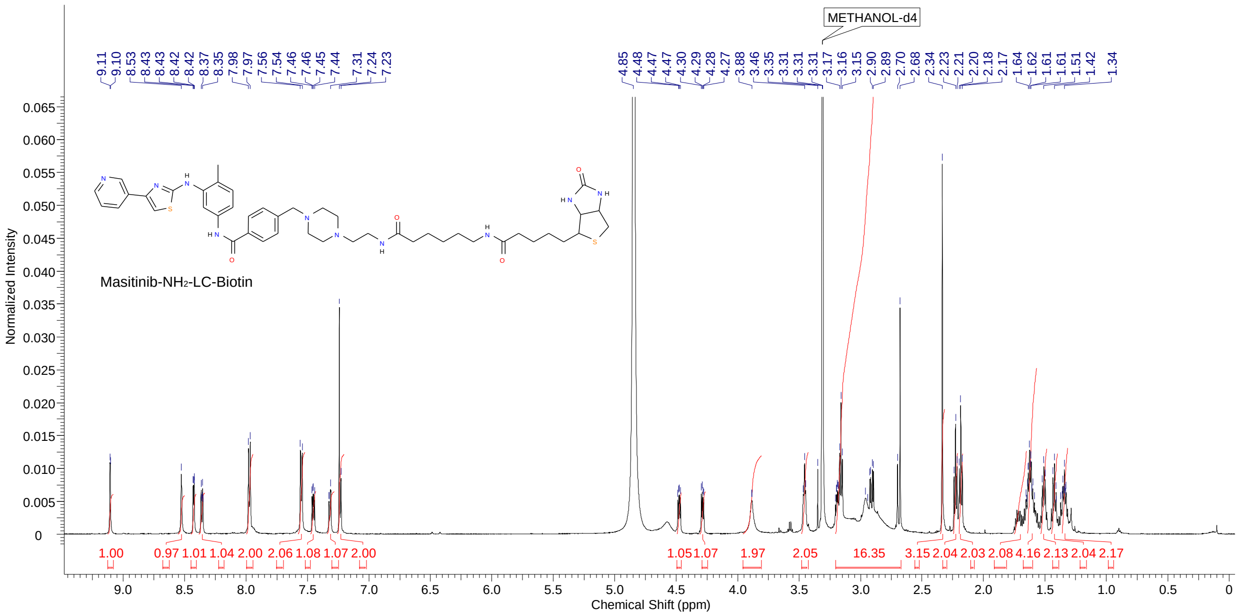

b

^{13}C NMR (150 MHz, CD_3OD) Masitinib-NH_2-LC-biotin

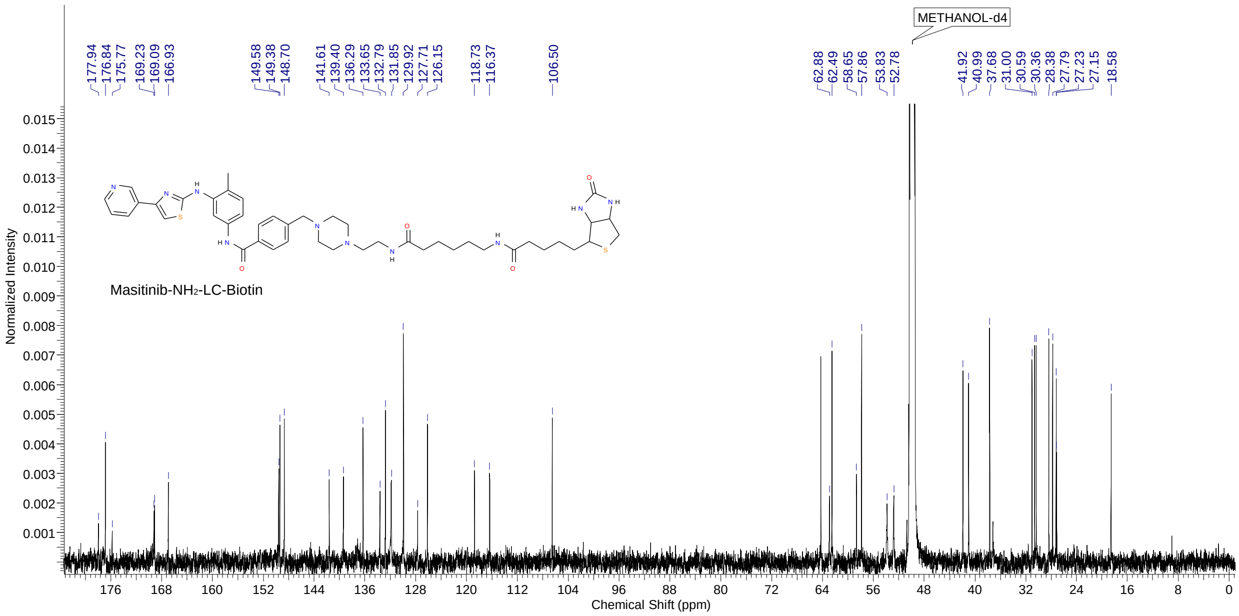

Supplementary Figure 14. Characterization of masitinib-NH_2-LC-biotin. (**a**) ^{1}H-NMR spectrum of masitinib-NH_2-LC-biotin in CD_3OD at 600 MHz and (**b**) ^{13}C-NMR spectrum of masitinib-NH_2-LC-biotin in CD_3OD at 150 MHz.

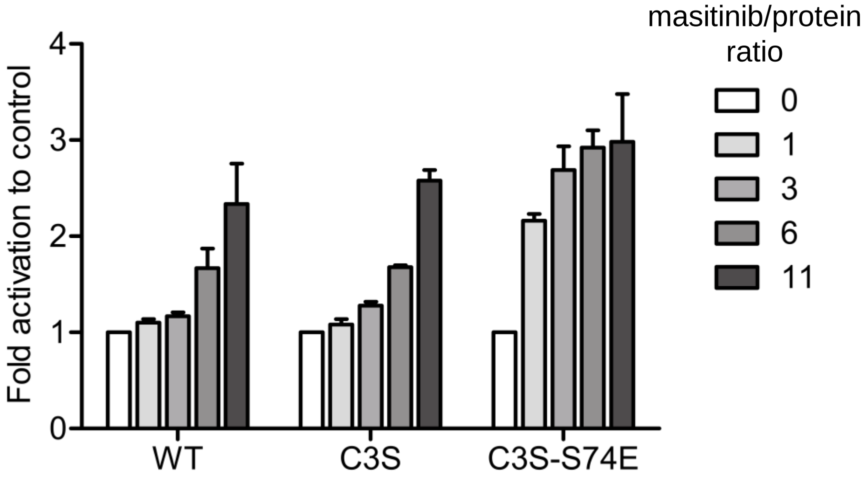

Supplementary Figure 15. Effect of masitinib on dCK-WT, dCK-C3S, and dCK-C3S-S74E enzymatic activity. Effect of masitinib on the phosphorylation of gemcitabine by dCK-WT, dCK-C3S, and dCK-C3S-S74E proteins in the presence of UTP. Masitinib (masitinib/protein ratios: 0, 1, 3, 6, 11) was able to enhance substrate phosphorylation by dCK-WT and mutants in the same concentration dependent manner. The experiments were performed in triplicates and data are presented as the mean ± s.d.

Figure 1d

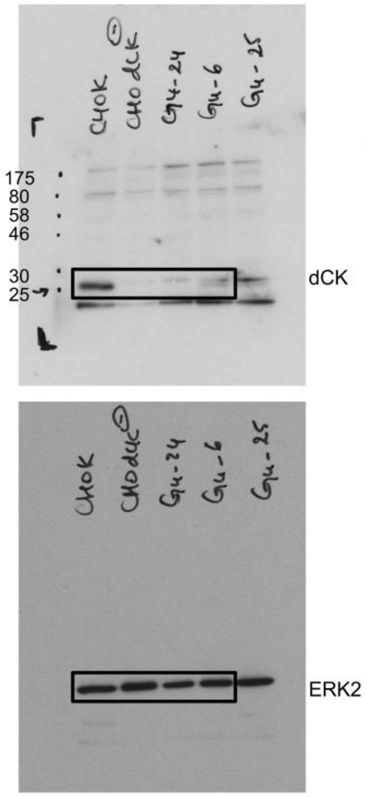

Supplementary Figure 3c

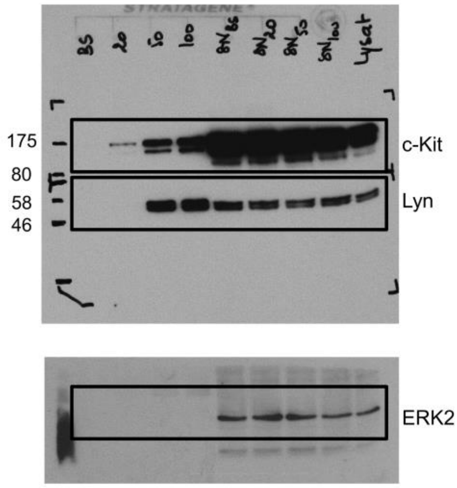

Supplementary Figure 5a

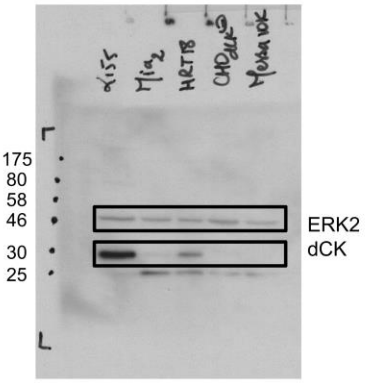

Figure 1c and Supplementary Figure 4

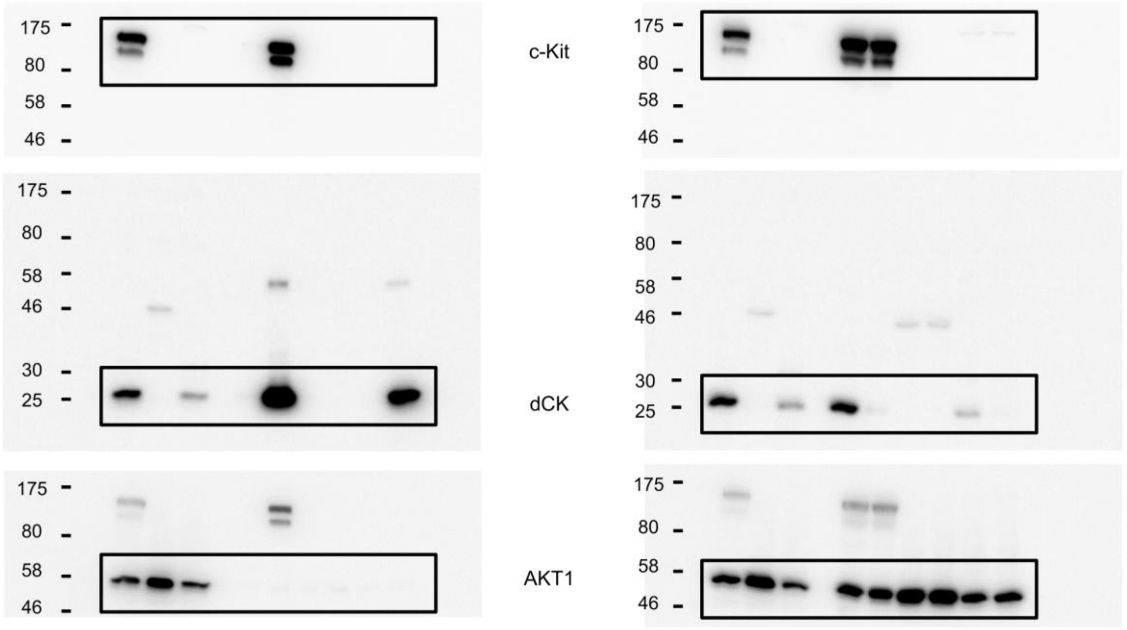

Supplementary Figure 16. Uncropped blots.

SUPPLEMENTARY TABLES

HMC1

				Fold change		P value (Anova)	
Accession	Description	Mass	emPAI	Data	Ranking	Data (x 10^{-3})	Ranking
DCK	**Deoxycytidine kinase**	30841	4,69	27,71	**3**	0,0818	**2**
LYN	**Tyrosine-protein kinase Lyn**	58993	2,13	11,18	13	0,684	22
ABL2	**Abelson tyrosine-protein kinase 2**	129289	0,08	9,54	18	6,22	101
KIT	**Mast/stem cell growth factor receptor Kit**	111163	0,09	1,97	75	6,09	97

HRT18

				Fold change		P value (Anova)	
Accession	Description	Mass	emPAI	Data	Ranking	Data (x 10^{-3})	Ranking
AGK	**Acylglycerol kinase, mitochondrial**	47564	0,31	12,12	12	4,06	375
DCK	**Deoxycytidine kinase**	30841	6,74	7,07	**27**	0,000686	**23**
LYN	**Tyrosine-protein kinase Lyn**	58993	0,31	5,51	41	0,00228	42
PRKDC	**DNA-dependent protein kinase catalytic subunit**	473749	0,73	2,49	105	0,000276	11

Supplementary Table 1. Kinases targets identified by reverse proteomic approach in HMC-1.1 and HRT18 cell lines.
The table lists the kinases identified from the mass spectrometry analysis that are statistically significant and that gave the largest fold change compared to the control beads. Protein mass and emPAI index (Exponentially Modified Protein Abundance Index) are included. emPAI index estimates protein abundance based on the number of peptides detected and the number of theoretically observed tryptic peptides for each protein[4]. Proteins are ranked by Fold change and P value. The fold change expresses the protein abundance between the two conditions; in the presence and absence of masitinib. P value is a measure of the statistical significance from ANOVA model.

	DRUG FREE	Masitinib 20 µM	Masitinib 50 µM	Masitinib 100 µM
V_{max}	530 ± 23	731 ± 29	1256 ± 37	2359 ± 72
K_m	227 ± 19	231 ± 21	289 ± 22	351 ± 30
R^2	0.9870	0.9903	0.9951	0.9905

Supplementary Table 2. Masitinib enhances phosphorylation of gemcitabine by dCK in the presence of UTP.
The V_{max} and K_m parameters calculated by fitting the experimental data to the Michaelis Menten model. The goodness of the fit is expressed by the R^2 value.

	dCK-C3S	dCK-C3S in complex with masitinib	dCK-C3S in complex with imatinib
**PDB ID**	5MQJ	5MQL	5MQT
**Data collection**			
X-ray source	ESRF		
Beamline	ID23-2	ID23-1	ID23-1
Space group	P 43 21 2	P 43 21 2	P 43 21 2
Cell dimensions			
a, *b*, *c* (Å)	92.69,92.69,338.08	88.33,88.33,342.53	93.3,93.3,342.71
α, β, γ (º)	90,90,90	90,90,90	90,90,90
Resolution (Å)	46.35-3.7 (3.833-3.7)	42.8-3.25 (3.366-3.25)	47.53-3.2 (3.314-3.2)
R_{merge}	0.1625 (1.021)	0.2677 (1.421)	0.1549 (1.175)
$I / \sigma I$	15.04 (3.15)	11.94 (1.45)	14.08 (1.60)
Completeness (%)	100 (99)	100 (100)	100 (100)
Redundancy	9.9 (10.2)	8.2 (8.6)	8.8 (8.8)
CC1/2	0.998 (0.911)	0.994 (0.832)	0.997 (0.744)
CC*	1 (0.977)	0.999 (0.953)	0.999 (0.924)
**Refinement**			
Resolution (Å)	46.35-3.7 (3.833-3.7)	42.8-3.25 (3.366-3.25)	47.53-3.2 (3.314-3.2)
No. reflections	16570 (1584)	22322 (2164)	25964 (2531)
R_{work} / R_{free}	0.2035 (0.2746) / 0.2611 (0.3610)	0.2318 (0.3580) / 0.2611 (0.3610)	0.2025 (0.2925) / 0.2582 (0.3232)
No. atoms			
Protein	7263	7332	7359
Ligand/ion	184	177	215
Water	0	18	15
B-factors			
Protein	131.18	97.39	87.59
Ligand/ion	130.7	111.39	104.66
Water	-	64.44	63.18
R.m.s. deviations			
Bond lengths (Å)	0.014	0.015	0.015
Bond angles (º)	1.91	1.89	1.90

Supplementary Table 3. Data collection and structural refinement statistics.
Statistics for the highest-resolution shell are shown in parentheses. A single crystal has been processed for each structure.

	BLI			
	K_D (μM)	k_{on} (1/Ms)	k_{off} (1/s)	t_R (s)
**Masitinib**	1.6 ± 0.4	1.2 ± 0.6 x 10^5	1.8 ± 0.4 x 10^{-1}	5.7 ± 1.2
**Imatinib**	4.7 ± 2.3	1.2 ± 0.2 x 10^5	3.9 ± 0.2 x 10^{-1}	2.6 ± 0.1
**DI-39**	0.54 ± 0.28	3.4 ± 2.7 x 10^5	0.45 ± 0.07 x 10^{-1}	23.0 ± 3.8

Supplementary Table 4. Kinetic parameters for dCK binding to masitinib, imatinib and DI-39 determined by Bio-Layer Interferometry (BLI).
BLI experiments were performed after immobilization of biotinylated dCK on SSA biosensors and binding/dissociation of the small compounds. K_D, k_{on} and k_{off} values obtained after the BLI experiment global fitting are listed.

Mutation	Primer	
**C9S**	forward:	CCGCCCAAGAGAAGC**TCC**CCGTCTTTCTCAGCC
	reverse:	GGCTGAGAAAGACGG**GGA**GCTTCTCTTGGGCGG
**C45S**	forward:	CCTTAAACAATTG**TCT**GAAGATTGGGAAG
	reverse:	CTTCCCAATCTTC**AGA**CAATTGTTTAAGG
**C59S**	forward:	CCTGTTGCCAGATGG**TCC**AATGTTCAAAGTAC
	reverse:	GTACTTTGAACATT**GGA**CCATCTGGCAACAGG
**S74E**	forward:	GAGGAACTTACAATG**GAA**CAGAAAAATGGTGGG
	reverse:	CCCACCATTTTTCTG**TTC**CATTGTAAGTTCCTC

Supplementary Table 5. Oligonucleotides used to mutagenize the human *dCK*.
Mutated codons are in bold.

SUPPLEMENTARY REFERENCES

1. Murphy, J.M. *et al*. Development of new deoxycytidine kinase inhibitors and noninvasive in vivo evaluation using positron emission tomography. *J. Med. Chem.* **56**, 6696-708 (2013).

2. Di Veroli, G.Y. *et al*. Combenefit: an interactive platform for the analysis and visualization of drug combinations. *Bioinformatics* **32**(18), 2866-2868 (2016).

3. Rix, U *et al*. Chemical proteomic profiles of the BCR-ABL inhibitors imatinib, nilotinib, and dasatinib reveal novel kinase and nonkinase targets. *Blood* **110**, 4055-4063 (2007).

4. Ishihama, Y. *et al*. Exponentially Modified Protein Abundance Index (emPAI) for Estimation of Absolute Protein Amount in Proteomics by the Number of Sequenced Peptides per Protein. *Mol. Cell. Proteomics* **4**, 1265-1272 (2005).
