# Supplementary material for: Dual protein kinase and nucleoside kinase modulators for rationally designed polypharmacology
Source: Nat Commun. 2017 Nov 10;8:1420. doi: 10.1038/s41467-017-01582-5 (PMC5681654; doi:10.1038/s41467-017-01582-5)
Supplement: Supplementary file 3 — Descriptions of Additional Supplementary Files [file 41467_2017_1582_MOESM3_ESM.pdf]

Descriptions of Additional Supplementary Files

File Name: Supplementary Dataset 1
Descriptions: Identification of masitinib targets by mass spectrometry on HMC-1.1 cellular lysates.

File Name: Supplementary Dataset 2
Descriptions: Relative quantification of masitinib associated proteins by mass spectrometry on HMC-1.1 cellular lysates.

File Name: Supplementary Dataset 3
Descriptions: Identification of masitinib targets by mass spectrometry on HRT18 cellular lysates.

File Name: Supplementary Dataset 4
Descriptions: Relative quantification of masitinib associated proteins by mass spectrometry on HRT18 cellular lysates.
